# Glancing-Angle Deposition of Nanostructures on an Implant Material Surface

**DOI:** 10.3390/nano9010060

**Published:** 2019-01-04

**Authors:** Nadine Ziegler, Christina Sengstock, Viola Mai, Thomas A. Schildhauer, Manfred Köller, Alfred Ludwig

**Affiliations:** 1 Institute for Materials, Faculty of Mechanical Engineering, Ruhr-University Bochum, Universitätsstraße 150, 44801 Bochum, Germany; nadine.ziegler@rub.de; 2 Surgical Research, BG University Hospital Bergmannsheil, Buerkle-de-la-Camp-Platz 1, 44789 Bochum, Germany; christina.sengstock@rub.de (C.S.); thomas.a.schildhauer@rub.de (T.A.S.); manfred.koeller@rub.de (M.K.); 3 Mathys Ltd. Bettlach, Robert Mathys Straße 5, CH-2544 Bettlach, Switzerland; viola.mai@mathysmedical.com

**Keywords:** sputtering, glancing angle deposition, nanostructures, antibacterial effect, cell compatibility

## Abstract

Cell-compatible and antibacterial surfaces are needed for implants, which frequently have complex and rough surfaces. Bio-inspired columnar nanostructures can be grown on flat substrates; however, the application of these nanostructures on clinically relevant, complex, and rough surfaces was pending. Therefore, a titanium plasma spray (TPS) implant surface was coated with titanium nano-spikes via glancing angle magnetron sputter deposition (GLAD) at room temperature. Using GLAD, it was possible to cover the three-dimensional, highly structured macroscopic surface (including cavities, niches, clefts, and curved areas) of the TPS homogeneously with nano-spikes (TPS+), creating a cell-compatible and antibacterial surface. The adherence and spreading of mesenchymal stem cells (MSC) were similar for TPS and TPS+ surfaces. However, MSC adherent to TPS+ expressed less and shorter pseudopodia. The induced osteogenic response of MSC was significantly increased in cells cultivated on TPS+ compared with TPS. In addition, Gram-negative bacteria (*E. coli*) adherent to the nano-spikes were partly destructed by a physico-mechanical mechanism; however, Gram-positive bacteria (*S. aureus*) were not significantly damaged.

## 1. Introduction

Titanium plasma spray (TPS) surfaces on dental or orthopedic implants are well established in medical technology and TPS-coated implants are clinically applied over many years. Titanium (Ti) is well known for its high biocompatibility and high corrosion resistance due to the formation of a thin and stable surface passivation layer (TiO_2_) [1,2,3,4], as well as its high specific strength [5]. In order to enhance the anchorage of a relatively smooth machined implant surface to bone, the implant surface can be modified by the deposition of a TPS-layer. An enhanced anchorage of bone to the implant is a determinant factor in osseointegration and consequently for long-term stability. In general, a TPS surface structure is very rough (macro-roughness of up to 240 µm micro-roughness approximately 40 μm) [6,7], as it is formed by overlapping droplets of solidified Ti [8], and is characterized by the occurrence of cavities, niches, clefts, and curved areas, resulting in a porous-like appearance. This special topography allows for an ingrowth of bone into the implant surface, as well as a direct structural and functional connection between living bone and the surface of a load-carrying implant (osseointegration) [9]. Such a connection reinforces the biomechanical interlocking of the bone with the implant [10]. Therefore, TPS surface layers are deposited, e.g., at high load bearing regions of implants for artificial joint replacement. TPS implants are frequently used in clinics, and additional nanostructures may further improve their performance by improving cell adherence and antibacterial properties. It is generally accepted that an implant surface topography, especially the surface roughness, influences the adherence of cells and bone response [11]. Besides the interaction of cells with macro- and micro-structured surfaces, the role of the nano-topography is being increasingly investigated, and several newly-developed advanced surface modifications were introduced to the medical implant system, focusing additionally on the nanoscale [12,13].

In the past, we fabricated Ti nano-spikes on thin, flat Ti film surfaces by glancing angle magnetron sputter deposition (GLAD). The height of these nano-spikes was in the range of 200 nm with separated columnar features [14]. Comparable nano-spike surfaces composed of biological material exist in nature on cicada wings, which were found to exert bactericidal effects on certain adherent bacteria by a physico-mechanical mechanism [15]. Our group demonstrated such an antibacterial effect for Gram-negative *E. coli* on GLAD-fabricated Ti nano-spikes on flat Si-SiO_2_ surfaces [16]. Similar structures have been sputtered on polished Ti6Al4V surfaces and show a strongly impaired adhesion of *S. aureus* combined with good cell response for osteoblasts [17]. The present study demonstrates the transfer of Ti nano-spikes’ nano-rough coating from flat substrates —which are generally used in thin film synthesis—to a clinically established, complex three-dimensional structure (TPS-layer) using GLAD. A uniform coating efficiency was achieved. The adherence of mesenchymal stem cells (MSC) as well as osteogenic differentiation of MSC were analyzed using the GLAD-coated compared to conventional non-coated TPS samples. In addition, structure-related antibacterial effects were tested using *E. coli* and *S. aureus*.

## 2. Materials and Methods

### 2.1. Sample Fabrication

**Figure 1:**
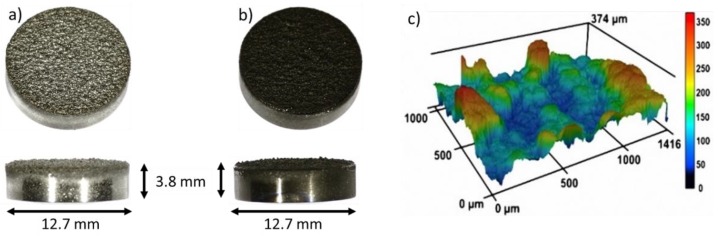
TiAl6V4 cylinders coated on one side with (**a**) 250 µm TPS and (**b**) with 250 µm TPS plus an additional GLAD Ti nanostructure (TPS+). In (**c**) a 3D image of the rough surface of the TPS sample measured with CLSM is shown.

The nanostructured coating was fabricated using GLAD on substrates which are an equivalent to the following clinically-used implant materials from Mathys Ltd. (Bettlach, Switzerland) (Figure 1a): TiAl6V4 cylinders (diameter = 12.7 mm, height = 3.8 mm) (ISO 5832-3:2016), thermal plasma spray coated with a 250 µm thick Ti layer (Ti grade 4 (ISO 5832-2:2018, ASTM F 67: 2013, ASTM F 1580:2012)). Within the TPS process, Ti powder particles are melted by plasma, and the droplets are accelerated to the substrate where they solidify. This process results in a typical high surface roughness that improves bone anchorage [18]. The TPS coating exhibits an average roughness of 45 ± 15 μm (according to DIN EN ISO 4288) and a porosity of 20–40% (according to ASTM F 1854). On these substrates, Ti nanostructures were deposited by GLAD in an AJA ATC-2200V sputter system (base pressure 2 × 10^−7^ Pa, sputter pressure: 0.666 Pa (Ar 6N)) at room temperature. GLAD is a special configuration of a magnetron sputter deposition process, where the sputter source (cathode) is arranged in an oblique angle—or even parallel to the surface—which leads to the growth of nanostructures over large areas [19,20]. The GLAD configuration comprised a 2-inch cathode (AJA A320-S-0874) with a Ti target (purity: 99.97%, diameter: 2″) aligned parallel (90° with respect to substrate normal) to the substrate, and rotating at 40 rpm. The substrate-to-target distance was 190 mm. The cylinders were positioned on a circular ring with a diameter of 60 mm in the sputter chamber to obtain the same substrate-to-target distance for each sample. Ti nanostructures were grown for 14,400 s with a power of 200 W, under a pressure of 7 × 10^−1^ Pa, and an Ar flow of 40 sccm. To guarantee the highest purity of the nanostructures, the Ti target was pre-cleaned for 600 s (power 200 W, Ar pressure 4 × 10^−1^ Pa, Ar flow 40 sccm). The resulting nanostructures were analyzed from a top view and in cross section using Scanning Electron Microscope (SEM) and Focused Ion Beam (FIB) (FEI Helios G4 CX dual beam workstation, Hillsborough, OR, USA). To analyze a larger cross-sectional area, one cylindrical sample was cut and ion polished with a Hitachi IM4000Plus and analyzed with SEM. Prior to cell experiments, all samples (TPS and TPS+) were cleaned using 70% ethanol, deionized water, and finally air dried at room temperature under sterile conditions. The roughness of the reference samples was measured with a confocal laser scanning microscope (CLSM, Keyence VK-X260K, Osaka, Japan).

### 2.2. Cell Culture

Human mesenchymal stem cells, obtained from Lonza Walkersville Inc. (Walkersville, MD, USA), were cultured in RPMI1640 (Life Technologies, Darmstadt, Germany), which was supplemented with fetal calf serum (10% FCS, Life Technologies, and L-glutamine (0.3 g L^−1^, Life Technologies). Cell culture procedures were followed as described previously [16]. Briefly, the MSC were maintained using 75 cm^2^ flasks (Falcon^®^, BD Biosciences, Heidelberg, Germany) at cell culture conditions (37 °C, humidified atmosphere containing 5% CO_2_). After removal of the cell culture medium, cell harvest was performed by addition of PBS containing 0.25% trypsin/0.1% EDTA (Sigma-Aldrich, Taufkirchen, Germany). After being washed twice with RPMI/10% FCS, the detached cells (1.5 × 10^4^ within 1 mL cell culture medium) were seeded onto TPS or TPS+ samples within wells of a 24-well cell culture plate (Falcon, Becton Dickinson) and cultured for an additional 24 h. Subsequently, samples were removed and washed twice with RPMI. Adherent cells were stained by fluorescence markers as described [16] using calcein-AM (Calbiochem, Schwalbach, Germany. Labelling of the cell nuclei was performed using a DAPI (Sigma-Aldrich, Taufkirchen, Germany). The cells were incubated with a final concentration of 300 nM DAPI in pure RPMI1640 for 5 min at 37 °C. Samples were washed twice with RPMI and cell adherence and morphology were analyzed by fluorescence microscopy (Olympus MVX10, Olympus, Hamburg, Germany). Fluorescence images were taken using F-view camera and Cell P software (Olympus). Image processing followed (Adobe Photoshop^®^ 7.0). This method allowed the qualitative visualization of cell morphology on the rough TPS topography and the quantification of adherent cells through the counting of the cell nuclei (from three predetermined regions (4.15 mm^2^) of three independent experiments).

### 2.3. Osteogenic Differentiation

For the analysis of osteogenic differentiation of MSC, 2 × 10^4^/mL RPMI/FSC were seeded onto TPS or TPS+ samples placed in wells of a 24-well cell culture plate and cultured for 24 h. The samples were then removed and transferred to wells of a new cell culture plate containing 1 mL RPMI/10% FCS or osteogenic differentiation medium (Life Technologies). Cell culture followed for 3 weeks and the osteogenic differentiation of the MSC on the samples was then analyzed by alizarin red staining. Briefly, the samples were washed 3 times with PBS and the cells were subsequently fixed with a 10% formaldehyde solution for 30 min. The cells were then washed again 3 times with distilled water and stained with 1% alizarin red S solution (Sigma-Aldrich) for 5 min. The staining was quantified by extraction with cetylpyridinium chloride (Sigma-Aldrich). Thereafter, the samples were washed again with distilled water and replaced headfirst to a new cell culture plate well. 200 µL of 10% cetylpyridinium chloride was then added and the plate was left shaking on a plate rotator for 40 min at room temperature. The supernatant was collected and measured at a wavelength of 570 nm using a Microplate-Reader (MRX Revelation, Dynex Technologies, Denkendorf, Germany). The extracted alizarin was quantified using a standard and given as µM. The relative mineralization was expressed as the percentage of extracted alizarin from mineralization of MSC on TPS (100%).

### 2.4. Bacterial Adherence

Bacterial stains were purchased from the Leibniz Institute German Collection of Microorganisms and Cell Cultures (DMSZ, Braunschweig, Germany). The analysis of bacterial adherence and cell morphology was performed using Gram-negative Escherichia coli DH5α (*E. coli*; DSMZ 6897) and Gram-positive Staphylococcus aureus (*S. aureus*; DSMZ 1104). Bacterial culture in a BHI broth solution followed as described [16]. TPS or TPS+ samples were placed on the bottom of the wells of 24 well plates (Falcon^®^, BD Biosciences). These wells were filled with 1 mL of *S. aureus* or *E. coli* solution containing 10^6^ bacteria. After 3 h at 37 °C, the bacterial solution was aspirated and the samples were gently rinsed twice with PBS to remove any non-adherent bacteria. Bacterial adherence was analyzed by fluorescence microscopy (Olympus BX61, Hamburg, Germany) after staining with Syto-9 (Molecular Probes, Eugene, OR, USA) according to the manufacturer’s instructions. Quantitative analysis of the fluorescent surface area was performed by phase analysis using cellSens software (Olympus). Images from three predetermined areas (each 46 mm^2^) were taken (magnification 3.15) from each sample of three independent experiments. For qualitative SEM analysis, samples were washed with PBS containing 5% glutaraldehyde (Sigma-Aldrich) overnight at 4 °C. After further washing with PBS, the bacteria were dehydrated in an ethanol series (30%, 50%, 70%, 80%, 90%, 96%, and 100%) for 5 min each. Subsequently, critical point drying with CO_2_ followed (K850, Quorum Technologies Ltd., Laughton, East Sussex, UK). Finally, TPS or TPS+ samples were coated with 15 nm Au-Pd (K500X, Quorum Technologies Ltd., Ashford, Kent, UK) and analyzed by SEM (FEI Helios G4 CX).

### 2.5. Statistical Analysis

Data are expressed as mean ± standard deviation (SD) of at least 3 independent experiments unless indicated otherwise. The Shapiro-Wilk test was used as a normality test. Quantification of adherent MSC was performed by the counting of DAPI stained cell nuclei of respective fluorescence images. Data obtained from three different experiments on three different surface areas of the same sample were statistically calculated using the Mann-Whitney rank sum test. Student’s *t*-test was used to analyze differences between osteogenic differentiation of MSC cultivated on the test samples and to determine the bacterial adherence to TPS and TPS+ samples. Significance levels were set to *p* < 0.05.

## 3. Results and Discussion

### 3.1. Formation of Nano-Spikes on the TPS Surface

**Figure 2:**
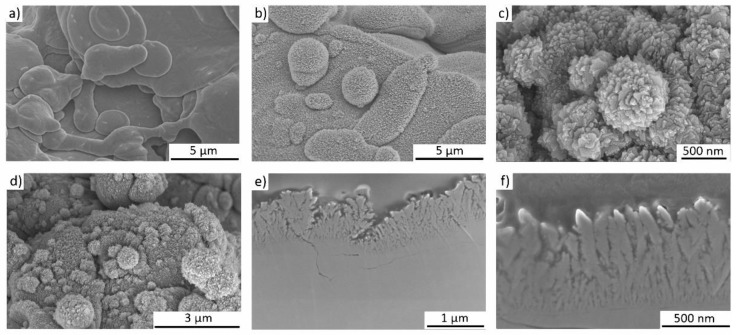
Representative SEM images of the implant surface (**a**,**d**) of the reference TPS sample with microscale roughness. In (**b**–**d**) SEM images of TPS+ samples with the additionally nanostructured surface after GLAD deposition are shown. The homogeneity of the nano-spikes over a wide area from the top view is shown in (**b**,**d**) and in greater detail sharp nanospikes are shown on solidified spherical droplets (**c**). In (**e**,**f**) images of the cross sections of the TPS+ samples with columnar-like structures are shown.

The complex surface topography of TPS samples (Figure 1a) was coated with an additional Ti nanostructure, with the aim to achieve hierarchically structured samples (TPS+, Figure 1b); i.e., the microscale rough features of the TPS samples (Figure 1c and Figure 2a) should be completely covered with nano-spikes. The Ti nanospikes consist of a volume of pure Ti, passivated by a thin TiO_2_ layer (typically in the range of several nanometers), which forms generally on Ti or Ti alloy surfaces exposed to ambient conditions [1,2,3,4]. As the nanospikes are deposited at room temperature, a very thin oxide layer is expected so that the relevant biointerface is a TiO_2_ surface. The growth conditions during GLAD on the rough TPS surface are complex, due to the high surface roughness and rotation of the substrates. Hence, the evolving microstructure cannot be estimated via structure zone diagrams for conventional deposition [21] and GLAD [22]. Therefore, experiments had to be conducted to verify if nanostructures can be synthesized on rough samples by GLAD (TPS+). Figure 1b shows a homogenous dark color of the TPS+ sample surface which already indicates less light reflection due to the nanostructured coating, which is present over the whole macroscopic sample. Figure 2b,d prove this through overview SEM images of the nanospikes, homogenously covering the rough TPS surface (Figure 2a).

Although the topography of the rough TPS surface is very complex, the results show that homogeneous nano-spikes can be obtained (see Figure 2b–d). Shadowing effects from the microscale roughness of the implant substrate are not observed, and consequently the sample surface is fully covered with nano-spikes. Eight samples were simultaneously coated with nano-spikes which were identical on all samples, showing that the GLAD process is suitable for coating large areas—as is needed for implants—with nanostructures. The homogeneity and the tip shape of the nanostructures is shown for the extreme case of deposition on solidified melt droplets in Figure 2c: even these spherical structures are homogeneously covered with sharp nano-spikes. In Figure 2e,f SEM images of a cross sectional sample are shown in a broad view and close up. These images visualize the homogeneity of the nanostructured coating and the sharp tips of the nano-spikes.

### 3.2. Biological Properties of the TPS+ Surface

#### 3.2.1. Cell-Compatibility

**Figure 3:**
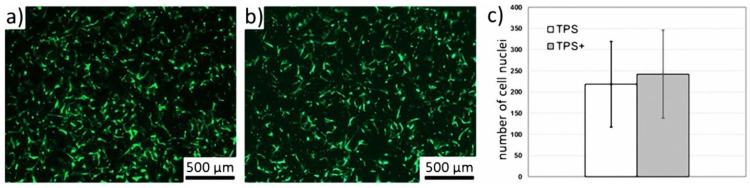
Representative fluorescence images of MSC cultured on (**a**) TPS and (**b**) TPS+ after 24 h incubation. The samples were stained with calcein-AM. (**c**) Quantification of adherent MSC on TPS and TPS+ samples. The quantification was performed by counting cell nuclei after staining with DAPI from respective fluorescence images.

It is well-known that bone response is influenced by surface topography [12]; thus, nanostructuring receives more and more attention to obtain a possible cell-instructing surface. These approaches reach from osteoblast differentiation up to structure-related antibacterial properties [23,24,25]. Our approach to coat a topographical complex TPS surface structure with an additional nanostructure covers both aspects. The nanostructured TPS+ surfaces should be tissue cell compatible and allow for good cell adherence. To compare the adherence of tissue cells to the TPS and the TPS+ samples we used human mesenchymal stem cells (MSC) as an experimental in vitro model. MSC are classical pre-tissue cells which are neither transformed cell lines nor immortalized cells but represent primary cells which can be cultured over several passages. Furthermore, MSC are found in different tissues such as bone marrow, fat, or muscle [26], and this cell type is intimately involved in tissue regeneration and tissue repair. Due to their high differentiating capacity, these cells represent an optimal cellular model to study differentiation [27]. Previously, we have shown that MSC adhered to Ti and TiO_2_ nano-spikes and were able to spread on these nanostructured thin films comparable to dense thin films [16]. For quantitative analysis, cells have been stained with calcein-AM and subsequently a calculation of the cell-covered surface was performed [16]. However, on the TPS samples the adherent MSC do not spread like that, but rapidly adapt to the local three-dimensional surface topography (see Figure 2), which does not allow quantification of the cell-covered surface. Therefore, cell nuclei were stained with DAPI and digitally counted using fluorescence images (mean number of three predetermined regions of interest for each image). Figure 3a,b show that there was no significant difference (*p* = 0.33, *n* = 3) in the MSC adherence after 24 h on TPS compared to TPS+ samples.

**Figure 4:**
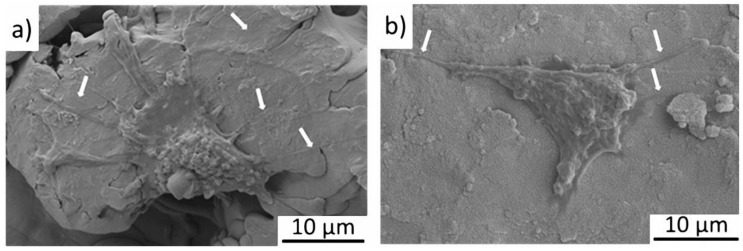
SEM images of adherent MSC on (**a**) the TPS structure and (**b**) the TPS+ structure. White arrows indicate pseudopodia of the cells.

However, a more detailed view on single adhered cells by SEM revealed a difference in the formation of pseudopodia (cytoplasmatic extensions) of the MSC on the different surfaces. Figure 4 shows that MSC adherent to TPS generate long pseudopodia on the relatively flat surface (a); in contrast, MSC on TPS+ form short pseudopodia, however less in number (b). It was reported that cells cultured on nanopatterned topographies displayed fewer pseudopodia compared with cells on flat surfaces [28]. The reason for this differing cell behaviour is not clear. Generally, pseudopodia are formed in order to adhere better to the surface, as well as to support the spread and migration of the cells [29]. It is possible that the flatter TPS surface at the nanoscale might favor cell migration in contrast to the nanostructured TPS+.

**Figure 5:**
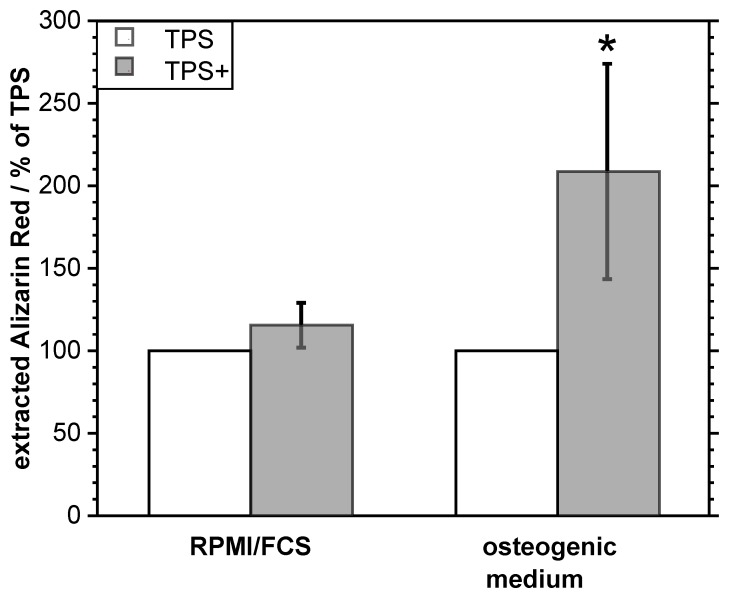
Osteogenic differentiation of MSC cultivated on TPS and TPS+ for 21 d in RPMI/FCS medium and in osteogenic medium. Extracted Alizarin Red S (TPS-RPMI/FCS 169 µM; TPS-osteogenic medium 1.741 µM) was normalized to the respective values obtained from TPS samples (100%). * *p* < 0.05 (*n* = 2).

To analyze the influence of the additional nanostructure on the TPS samples on the osteogenic differentiation of adherent MSC, the TPS and TPS+ samples were loaded with MSC and incubated for 21 d either in RPMI/FCS medium or an osteogenic medium. The formation of calcium phosphate matrix was quantified by dye extraction after staining with Alizarin Red S. Figure 5 shows that there was no spontaneous osteogenic differentiation of cells on TPS+ compared to TPS. However, after induction of an osteogenic differentiation by an osteogenic medium, a significant increase in the osteogenic response of MSC on the TPS+ surfaces occurred. These data indicate that a TPS+ surface might promote ongoing osteogenic processes.

In general, the micro- and nanostructures of an implant surface contribute to osteoblast responses and have a large impact on the resulting implant osseointegration [11,24]. There are several reports which have shown that the surface nano-topography or nano-roughness promote the osteogenic differentiation of MSC even in the absence of added osteogenic supplements [24,28,30]. We did not observe an enhanced osteogenic differentiation of cultivated MSC without an additional osteogenic stimulation in our in vitro experimental setup. However, an ongoing osteogenic process, which may be initiated in vivo after implantation by physiologic regeneration processes, may be promoted by the nanostructure on a TPS+ surface. One explanation might be better calcium phosphate or protein deposition on the TPS+ due to the special nanotopography and the enhanced surface area. It is known that the surface nano-roughness favors downstream signaling pathways via transmembrane receptors that recognize these changes in the surface [31]. However, this needs to be analyzed in more detail.

#### 3.2.2. Antibacterial Properties

In addition to the desired improvement of tissue and bone ingrowth, the complex 3D-structure of a TPS surface with many niches and cavities may offer better hideaway areas for bacteria compared to a flat and smooth surface. We have already demonstrated that structurally comparable Ti nano-spikes generated by GLAD on flat Si-SiO_2_ samples induced an antibacterial effect [16]. It was shown that the cell envelope of adherent Gram-negative *E. coli* were partly ruptured by physico-mechanical interaction of the bacterial cell wall with the nano-spikes. In contrast, Gram-positive *S. aureus* were not affected by such a nanostructured surface [16]. The present study clearly shows that coating of TPS with a similar nano-spike surface induced similar antibacterial effects on *E. coli* but generally not on *S. aureus*.

**Figure 6:**
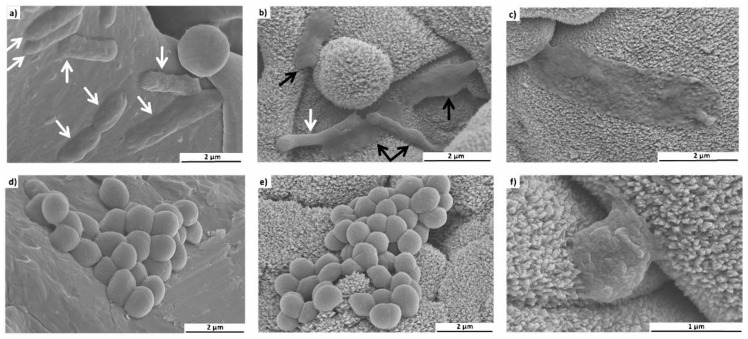
Representative SEM images of *E. coli* (**a**–**c**) and *S. aureus* (**d**–**f**) adherent to TPS (**a**,**d**) and nanostructured TPS+ (**b**,**c**,**e**,**f**) samples. The white arrow indicates morphologically intact *E. coli*; black arrows, damaged *E. coli*.

A detailed investigation by SEM (Figure 6) demonstrated morphologically intact *E. coli* adherent to TPS (a). On TPS+ damaged *E. coli* are detectable (b, black arrows) and also completely collapsed *E. coli* (c, black arrow). However, morphologically intact cells can also be identified (b, white arrow). The *S. aureus* were relatively unaffected by nanostructured TPS+ just as well as by the TPS. Typical grape-like colonies could be found on both surfaces (d,e). However, in rare cases individual *S. aureus* found in a narrow cleft were damaged (f). As we have shown previously, a possible explanation might be that ongoing cell divisions within the narrow cavities push the cell walls onto the spiky surfaces, leading to structural cell damage [32].

**Figure 7:**
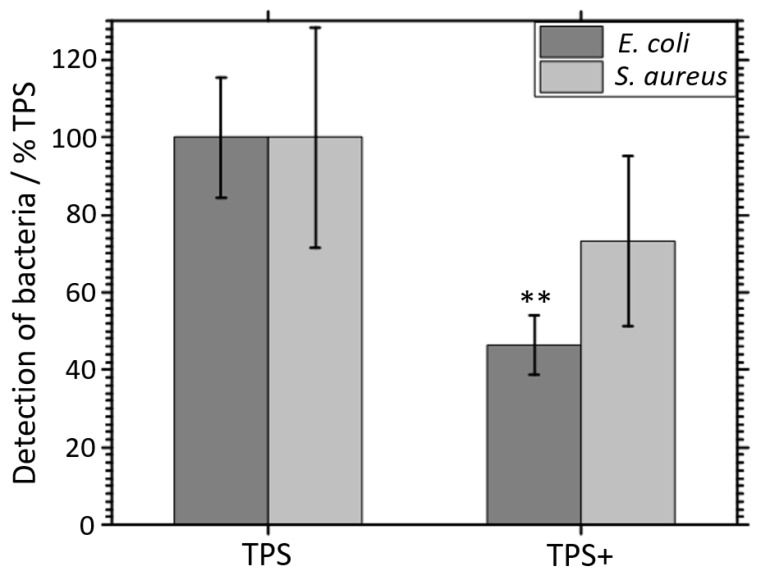
Quantification of adherent *E. coli* and *S. aureus* on TPS and TPS+ samples. Quantification was performed by image processing (phase analysis) after fluorescent staining of the bacteria. ** *p* < 0.01, *n* = 3).

Quantification of the bacterial adherence was performed by digital image processing after fluorescence staining. As is shown in Figure 7, the detection of *E. coli* was significantly lower (*p* < 0.01, *n* = 3) on the nanostructured TPS+ samples compared to the TPS samples. In contrast, the detection of *S. aureus* did not differ between both surface types (*p* = 0.27, *n* = 3).

The observed antibacterial effect induced by the nano-topography is obviously not sufficient to contain a bacterial infection in a clinical situation where bacteria may hide in cavities and clefts of the TPS without direct contact to the implant material. However, the nano-spikes may reduce the number of adherent bacteria and therefore may reduce the risk of biofilm formation. Currently, efforts are being made to extend the material-related antibacterial effect of such nano-spikes to Gram-positive bacteria, which play a major role in implant-associated infections, by decoration with antibacterial active Ag.

## 4. Conclusions

This study demonstrated the effective transfer of biologically inspired nanostructures on samples of clinically-used TPS implant surfaces. Although TPS surfaces are highly structured (including three-dimensional cavities, niches, clefts, and curved areas) their whole surface can be homogeneously covered by nano-spike structures using GLAD at room temperature. This nanostructured surface promotes the osteogenic response of mesenchymal stem cells and exerts structure-related antibacterial effects on Gram-negative bacteria (*E. coli*). Such nanostructures may be further improved to develop new implant topographies which combine antibacterial and osteopromotive activities.
